# Synergetic effect of essential oils and calcium phosphate nanoparticles for enhancement the corrosion resistance of titanium dental implant

**DOI:** 10.1038/s41598-024-52057-9

**Published:** 2024-01-18

**Authors:** Heba Tarek Zaher, Mahmoud A. Hefnawy, Shymaa S. Medany, S. M. Kamel, Sahar A. Fadlallah

**Affiliations:** 1 Chemistry Department, Faculty of Science, Cairo University, Giza, 12613 Egypt; 2 Biotechnology Department, Faculty of Science, Cairo University, Giza, 12613 Egypt; 3 Oral Biology, October University for Modern Sciences and Art, MSA University, Giza, Egypt

**Keywords:** Biotechnology, Health care, Medical research, Materials science

## Abstract

Calcium phosphate (CaPO_4_) coating is one of various methods that is used to modify the topography and the chemistry of Ti dental implant surface to solve sever oral problems that result from diseases, accidents, or even caries due to its biocompatibility. In this work, anodized (Ti-bare) was coated by CaPO_4_ prepared from amorphous calcium phosphate nanoparticles (ACP-NPs) and confirmed the structure by X-ray diffraction (XRD) and Fourier-transform infrared spectroscopy (FT-IR) techniques. Ti-bare was coated by prepared CaPO_4_ through the casting process, and the morphology of Ti/CaPO_4_ was characterized by scanning electron microscope (SEM) where the nano-flakes shape of CaPO_4_ and measured to be 60 ~ 80 nm was confirmed. The stability of Ti-bare and coated Ti/CaPO_4_ was studied in a simulated saliva solution using electrochemical impedance spectroscopy (EIS) and linear polarization techniques to deduce their corrosion resistance. Furthermore, three essential oils (EO), Cumin, Thyme, and Coriander, were used to stimulate their synergistic effect with the CaPO_4_ coat to enhance the corrosion resistance of Ti implant in an oral environment. The fitting EIS parameters based on Rs [R_ct_C]W circuit proved that the charge transfer resistance (R_ct_) of Ti/CaPO_4_ increased by 264.4, 88.2, and 437.5% for Cumin, Thyme, and Coriander, respectively, at 2% concentration.

## Introduction

Millions of people worldwide are affected by dental caries, a chronic disease with high prevalence. The first stage of dental caries appears as white spots that form non-cavitated lesions, and the second stage is carious cavities. Once dental caries occurs, it will be hard to reverse or stop it from progressing. The ultimate objective is to stop the caries process using non-invasive methods at that white spot lesion stage. There are different conventional techniques for caries removal, but the most famous one is Chemo-mechanical caries removal. After the caries get removed, composite material to fill the teeth is necessary. A composite that can strongly bond with the teeth to form the best adherence ability is considered one of the important abilities needed for a successful restoration process^1–4^. Caries may appear during orthodontic treatments, from the enamel to leak—-cavitation that leads to loss of minerals, primarily calcium and phosphorus loss. Therefore, special composite can be used on the labial surfaces or banded teeth through enamel decalcification and remineralization^5^. Amorphous Calcium phosphate (ACP) is a promising compound that can release calcium and phosphate for the prevention and treatment of decalcification. At the same time, ACP nanoparticle (ACP-NPs) have recently attracted much attention because of their outstanding performance and unique area that enhances the stability of implant metal in the oral environment^6^.

Titanium and its alloys are used as dental implants to solve the severe oral problems that result from diseases, accidents, or even caries due to their fascinating properties like excellent biocompatibility, high corrosion resistance, acceptable strength-to-weight ratio, low elastic modulus, and low density^7–11^. Based on the chemistry of the titanium surface, which is naturally oxidized and covered with a thin layer from titania (TiO_2_), through the anodization process, the thin TiO_2_ layer is enlarged in its thickness to enhance the biocompatibility of Ti implant in physiological solution^12^. TiO2 layer is easily hydrated when exposed to an aqueous medium, and the developing hydroxyl functional group will improve the chemical activity toward other ions such as phosphate and calcium^13^. Furthermore, calcium phosphate was reported as an effective coat for anodized Ti metal and its alloys^16–18^ and other biomaterial metals such as Mg, Zn, Sr, Ca, Ag, and Zr^14–17^, due to the obtaining of morphology and chemical structure similar to natural bone.

Plants contain compounds like carotenoids, phenols, vitamins, and flavonoids that reduce agents^18–20^. Plant extract products, such as essential oils, are used in tiny concentrations to protect the surface of metals from corrosive environments. The plant extracts can also be considered site blockers or adsorption site blockers because of their ability to absorb on the metal surface. Herbal extracts can be used as an early intervention strategy to manage caries, using as grape seeds, cinnamon oil, tea tree oil, and garlic extract are receiving more attention for their use as phytotherapy is preferred due to their biocompatibility and effectiveness. These extracts have been found to have strong antimicrobial and preventive effects on synthetic antibiotics^21^. In another perspective, several natural products were reported in the literature as effective green protective solutions, such as Delonix *regia*; rosemary leaves extracts that inhibited the corrosion of aluminum, natural honey for inhibition of copper corrosion, and African bush pepper; neem leaves; *Carica papaya* leaves extract for inhibition of mild steel corrosion^22–27^. However, the organic compounds' effectiveness regards the molecular chemical formula and its related physicochemical properties of the compound, like the presence of donor/ acceptor atoms and the extended functional groups^28,29^. Additionally, these natural compounds support the stability of the metal by adsorption or forming protective barrier films on the metal surface^22^.

Cumin (Cuminum cyminum L.) is a member of the Apiaceae family that spread through Mediterranean region, Middle East, and India. It considered as traditional medicine due to various active ingredients like terpenes, phenols, and flavonoids^30–32^. Thus, the presence of phenolic compound in Cumin leads to enhance the anti-corrosion ability as reported for different metals and alloys like aluminum, copper, and steel^33–35^

Thyme (Thymus vulgaris L.) is considered as a member of the Lamiaceae family, Whereas the Thyme is widely spread economically in North America, Europe, and North Africa^36^. Due to the presence of aromatic compound, the Thyme plant extract was observed to have high effectiveness toward corrosion of different Alloys like mild steel, and 304 steel^37–39^.

Coriander (Coriandrum sativum L.) contains essential oils in its leaves, flowers and seeds. Thus the coriander is considered as member of the Apiaceae family, is among most widely used medicinal plant, possessing nutritional as well as medicinal properties. The Coriandrum is widely reported in literature as green corrosion inhibitor for several metals and alloys like aluminum, mild steel, 6063 aluminum alloy, and carbon steel^40–43^.

Electrochemical impedance spectroscopy (EIS) is one of the most powerful techniques widely used to estimate the electrochemical behavior of the metals in the electrolytic solutions to characterize various electrochemical systems^44–50^. Thus, it shows and deduces the contribution between the metal surface and the electrolytic solution under electrochemical conditions. Furthermore, it can be used for simulating the dynamics of active mobile charge in the interface regions by fitting the out-put EIS data into different impedance parameters^51–58^.

In the present work, a new comparative study was performed on Ti- bare and coated Ti/CaPO_4_ samples to deduce the positive impact of CaPO_4_ under the synergistic effect of essential oils, where they act together for enhancement of the corrosion resistance of Ti dental implant in simulated oral solution. Ti/CaPO_4_ sample was soaked in the saliva solution for different intervals periods of up to 14 days to determine the change in charge transfer resistance, corresponding to the corrosion resistance, with time. Furthermore, Cumin, Thyme, and Coriander oils were added to the artificial physiological solution to deduce the synergistic relation of each with the CaPO_4_ coat and its positive impact on the implant surface. Also, the linear polarization technique was employed to measure the impact of oil on the corrosion process, and the corrosion rates of Ti-bare and coated Ti/CaPO_4_ were studied. Otherwise, the comparison between different oils.

## Experimental work

### Anodization

Through the anodization process, the layer of TiO_2_ was formed as follows: the Ti-foil with an area of 1 cm × 1 cm and thickness of 1mm (99.99% metal basis, Sigma Aldrich) was used as the starting material. First, the Ti degreasing was performed by sonication in a mixture of acetone, isopropanol, and methanol. Then, the foil was washed with deionized water and dried in nitrogen steam. The Ti sample was connected at the positive pole of the power supply and immersed in a solution of 1.0 M H_3_PO_4_ + 0.8 wt.% NaF doing as oxidants in the electrochemical cell at a constant potential of 20 V for 30 min. Finally, the sample after anodization was cleaned by immersion several times in deionized water and dried overnight in an oven under a nitrogen atmosphere, and then the Ti-bare sample was ready. The main goals for surface treatment through anodization are enhancement of bioactivity, biocompatibility, and corrosion resistance for dental applications.

### Preparation of ACP-NPs

Potassium hydrogen phosphate was dissolved in deionized water at room temperature, along with the addition of calcium nitrate to form a P-precursor Ca-precursor solution with concentrations 0.040 M and 0.036 M, respectively. The pH value of the solution was fixed and adjusted at 8.0 by appending ammonium hydroxide solution (0.1 M) while continuously stirring at room temperature (25 °C) until a white slurry formation was achieved. The solution was centrifuged at 7000 *rpm* for 10 min and washed with distilled water three times. The prepared calcium phosphate precipitate was washed with acetone three times and dried at room temperature for 24 h; they were later stored under a vacuum for chemical structure characterization by XRD & FT-IR and further electrochemical studies.

### Electrochemical measurements of prepared Ti/CaPO_4_

To produce a new modified Ti implant surface by ACP-NPs and subsequently evaluate its electrochemical behavior, a working electrode was utilized in the experiment. It consisted of an anodized sample Ti-bear with a surface area of 0.0625 cm^2^ and a thickness of 1 mm. Initially, the surface underwent a polishing process utilizing a gentle emery paper, followed by a thorough rinse with double distilled water and ethanol.

The ACP-NPs were prepared from 50 mg calcium triphosphate and suspended in 1 mL of ethanol. The 100 µL of suspension was cast on the anodized Ti-bare surface. Finally, the surface was dried overnight. The samples were nominated by Ti/CaPO_4_ when not exposed to the essential oils, but when exposed to EO named based on the type of the oil used as Ti/CaPO_4_-L1, Ti/CaPO_4_-L2, and Ti/CaPO_4_-L3 for Cumin, Thyme, and Coriander oils, respectively. The essential oils were supplied by local brand “Harraz” the concentration of pure essential oil is ~ 100%.

EIS and polarization results were achieved using Autolab PGSTAT128N. The electrochemistry software NOVA fitted the impedance spectrum (Version 2.1, Metrohm Autolab, Utrecht, Netherlands). A three-electrode cell was used, utilizing distinct surfaces as working electrodes. The reference and auxiliary electrodes employed were Ag/AgCl/KCl (saturated) and Pt wire, respectively.

The artificial saliva was prepared as mentioned in Table 1. That the pH was adjusted to 7.4.

**Table 1:** The composition of artificial saliva solution.

Composition	Potassium chloride	Calcium chloride dihydrate	Sodium chloride	Potassium phosphate monobasic	Sodium phosphate dibasic	Potassium bicarbonate	Potassium thiocyanate	Citric acid
Conc. (g/L)	0.720	0.220	0.600	0.680	0.866	1.500	0.060	0.03

## Result and discussion

### Surface characterization

**Figure 1:**
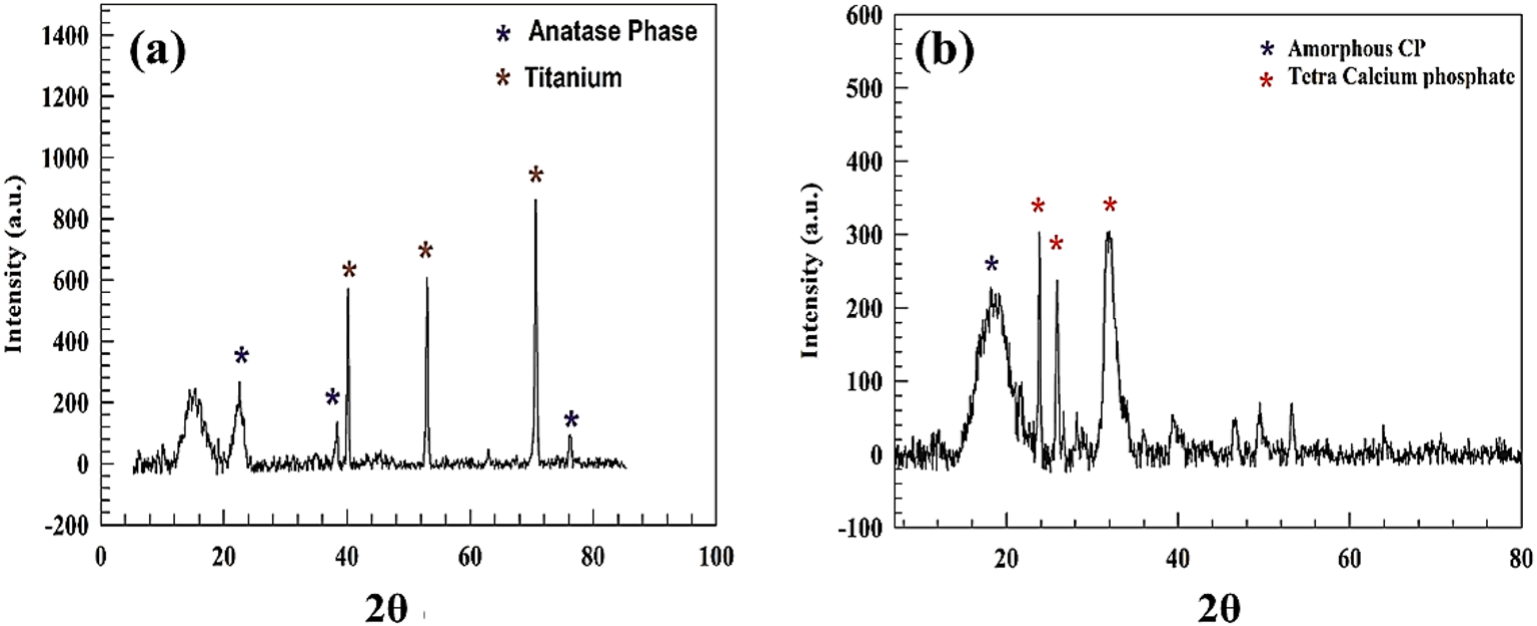
XRD chart of (**a**) Anodized Ti (Ti-bare), (**b**) Amorphous CaPO_4_ nanoparticles ACP-NPs.

The chemical structure of both samples, anodized titanium Ti-bare and coated Ti/CaPO_4_, were characterized by X-ray diffraction techniques. As represented in Fig. 1a, the XRD chart for anodized Ti surface. The characteristic peaks of titanium were observed at 2θ = 39.8. 52.3 and 71.1° for attributed to miller indices (100),(200), and (211) respectively^59,60^. Accordingly, TiO_2_ exists in crystal structure and point group of hexagonal and 6/mm, respectively. Furthermore, the anatase titania (TiO_2_) phase formed via anodization was observed in Fig. 1a, which has three peaks at 2θ = 22.9, 38.2, 76.3 for corresponding miller indices of (101), (004), and (211), respectively (JCPDS card no. 21-1272). The point group and crystal structure are recommended to be P4_2_/mnm and tetragonal, respectively. As represented in Fig. 1b, the calcium triphosphate was characterized by the peaks at 2θ =, 26.02, 31.6, and 32.3 o for corresponding miller indices (002), (211), and (300)^61,62^. Whereas the space group and crystal structure are recommended to be R3̅m and Trigonal, respectively. Furthermore, the crystal structure of calcium phosphate consisted of two inequivalent Ca^2+^ points. The Ca^2+^ is bounded to six equivalent O^2−^ atoms in the first site. Thus, the Ca-O bond lengths are equal to 2.46 Å. On the other hand, the second site of Ca^2+^ atoms is bounded to 10 coordinate geometry to 10 oxide atoms (O^2−^). Hence, the Ca-O bond is ranging 2.25–2.72 Å. At the same time, the P-O is represented in short and long bond lengths equal to 1.54 and 1.56 Å, respectively^63,64^.

**Figure 2:**
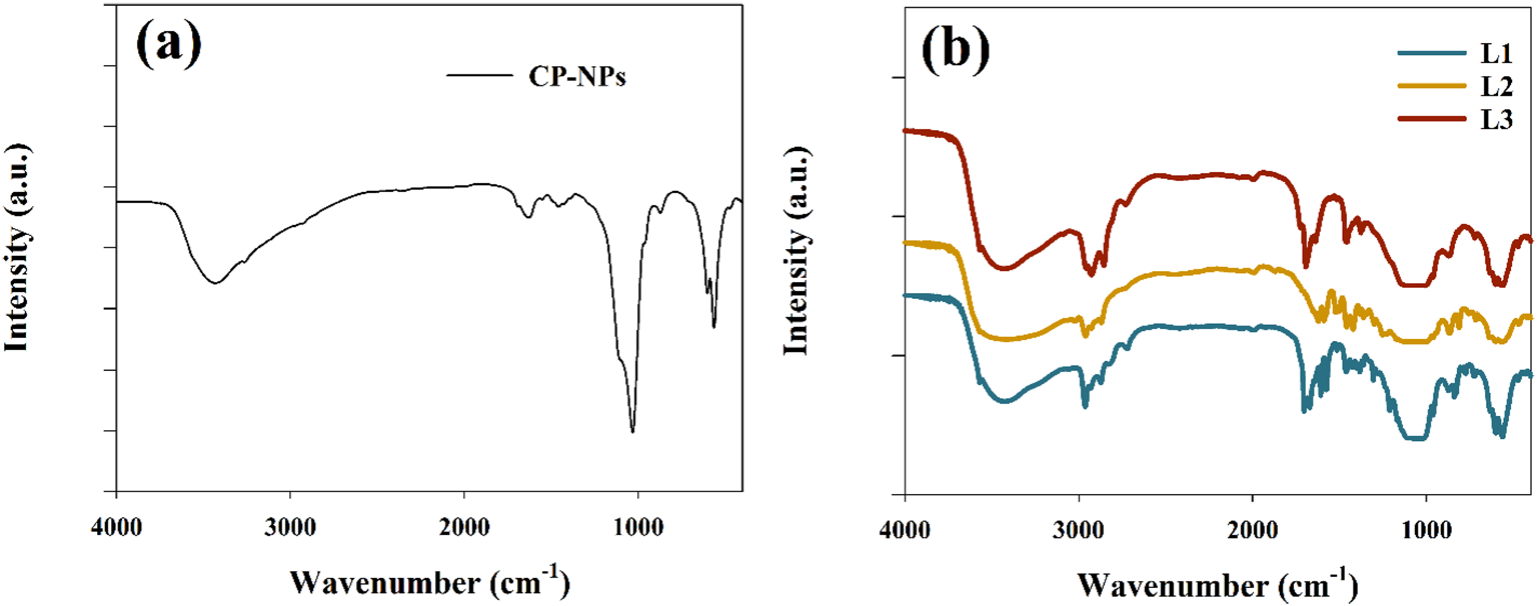
FT-IR- chart (**a**) Calcium Phosphate (CP-NPs), (**b**) Essential oils.

The bond stretching within the molecule of the prepared CaPO_4_ was studied by the FT-IR spectroscopy method to determine the functional groups. As illustrated in Fig. 2a. The peaks in the range of 3427 cm^−1^ and 1639.1 cm^−1^ refer to O–H stretching vibration modes of adsorbed water traces^65^. In addition, the peaks at 1034 and 563.3 cm^−1^ correspond to the orthophosphate group vibration modes^66^. Furthermore, the IR spectra for different essential oils were studied as represented in Fig. 2b. the peak observed at 3446 cm^-1^ corresponding to the O–H stretching in water molecule on the surface^44^. else, the peak at wavenumber equal to 2994, and 2820, and 1749 cm^−1^ attributed to the functional groups of (sp3)C–H, and –(O=C–H) and C=O respectively^67–69^.

**Figure 3:**
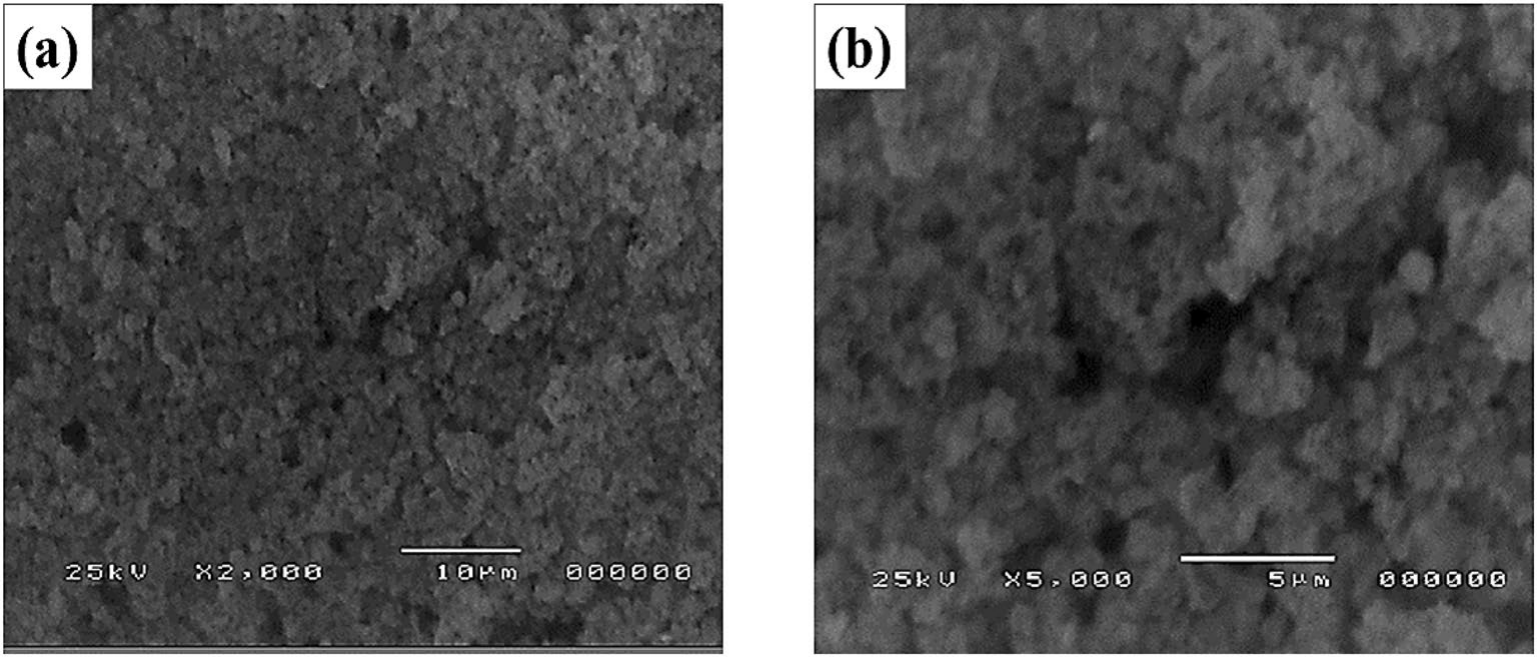
SEM Image of the amorphous CaPO_4_ on the anodized Ti surface.

The surface morphology of the CaPO_4_, which will deposit on the anodized Ti surface, was characterized by SEM, as represented in Fig. 3, at two different magnifications. As represented in Fig. 3, the nanosized CaPO_4_ particles were confirmed, and it was found to have a nano-flake shape and measured to be 60 ~ 80 nm. This figure illustrates more cavities on the surface, which increases the surface roughness, which is important to raise the biocompatibility of the Ti surface in saliva solution via increasing the corrosion resistance, as illustrated in the following sections. As we know, ACP is essential for the formation of mineralized bone and is used for bone substitutes^70^.

**Figure 4:**
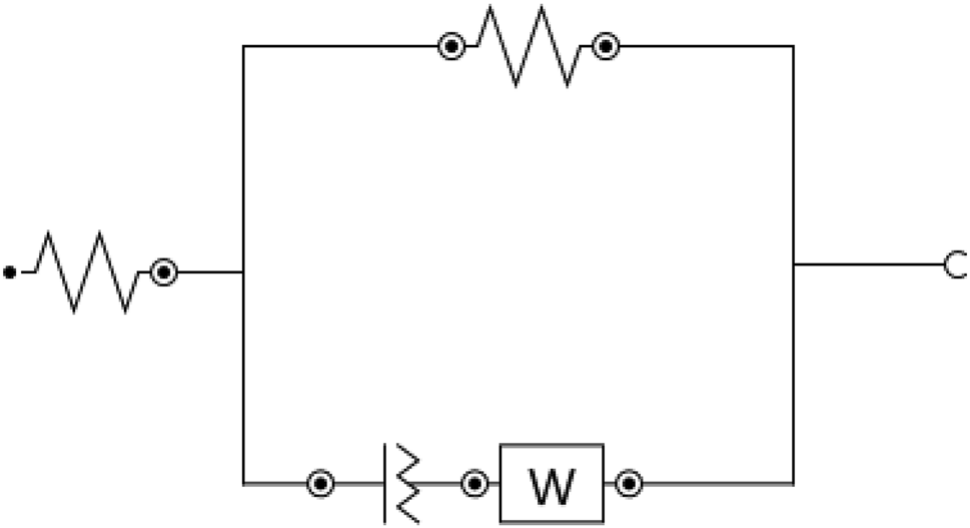
Fitting circuit of the EIS data.

In the following sections, the electrochemical behavior of the anodized Ti/CaPO_4_ samples in compared with Ti-bare metal was investigated through the electrochemical impedance studies to estimate the corrosion resistance of these samples towards the simulated physiological solutions which containing the concentrations of salts and ions like; Cl, Na, P that play a responsible role in changing on the stability of these surfaces. We will discuss in detail the interpretation of the EIS measurements for the coated Ti/CaPO_4_ sample with or without the effect of essential oils (EO) in comparison with the Ti-bare sample. During the fitting process, the EIS data were fitted using ANOVA software, and the fitted circuit is illustrated in Fig. 4. The fitting model consists of solution resistance (R_s_) connected in series with (R_c_//C), where R_c_ is a charge transfer resistance, and C is a capacitor element. The presence of a constant phase element regards the nationally formed anatase layer (TiO_2_), as reported in the XRD part. Furthermore, the constant phase element of EIS is mathematically like the capacitor component. Where the following equation can employ the impedance of CPE^71–73^:

1 $${\text{Z }} = \, \left( {{1}/{\text{Y}}_{{\text{o}}} } \right)/ \, \left( {{\text{J}}\omega } \right)^{\alpha } ,$$

where the proportional factor (Y_o_) is the CPE constant, the angular frequency is (ω) (in radians/sec), the imaginary number j^2^ =  − 1, and n is the CPE exponent ranges from zero to one.

**Figure 5:**
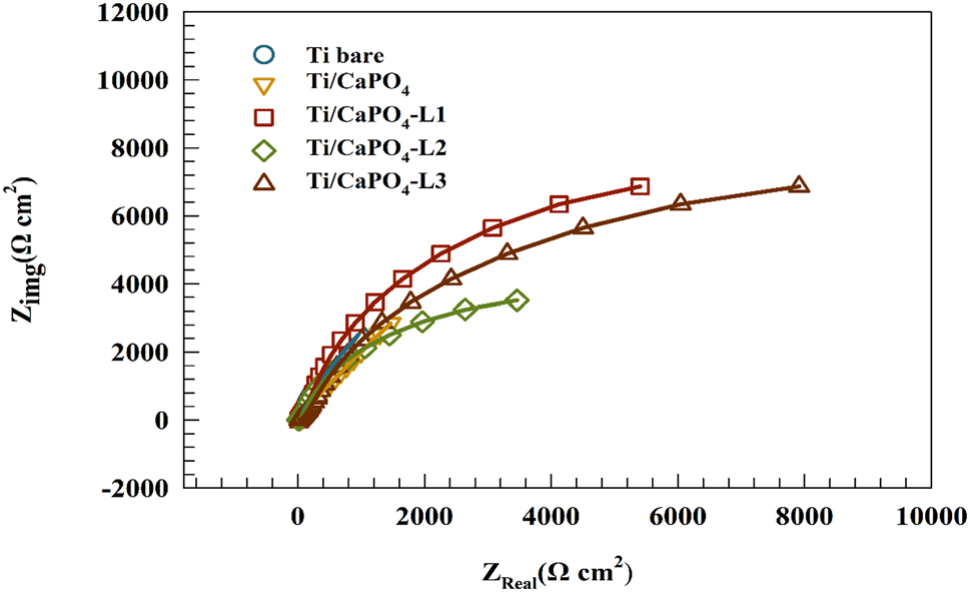
Comparison of different modified CaPO_4_ Ti surfaces in simulated saliva solution (i.e., Ti-bare, Ti/CaPO_4_, Ti/CaPO_4_-L1, Ti/CaPO_4_-L2, Ti/CaPO_4_-L3.

Given the EIS Nyquist spectra of the present samples after 3 h of immersion in simulated saliva solution containing EO, was represented in Fig. 5. We noticed that the EO (L1 or L2 or L3) plays a great role in raising the corrosion resistance of the anodized Ti/CaPO_4_. These results support our hypothesis that while the CaPO_4_ on anodized Ti acts as a bioactive coat, a synergistic effect emerges between the EO CaPO_4_, acting as a superior for the stability of Ti sample in the oral environment. In the following sections, we will be giving more evidence to confirm the synergistic relation between the CaPO_4_ on the surface and the EO in the solution to prove its positive impact on the corrosion resistance of Ti in simulated solutions. For this reason, we will discuss two categories of EIS measurements. The first one focuses on the study of the electrochemical behavior of Ti/CaPO_4_ in comparison to Ti-bare sample without the addition of EO in saliva solution for two periods, a short time of 240 min and a long time of 336 h In the second category, we study the effect of the addition of EO in the solution for the same two periods.

**Figure 6:**
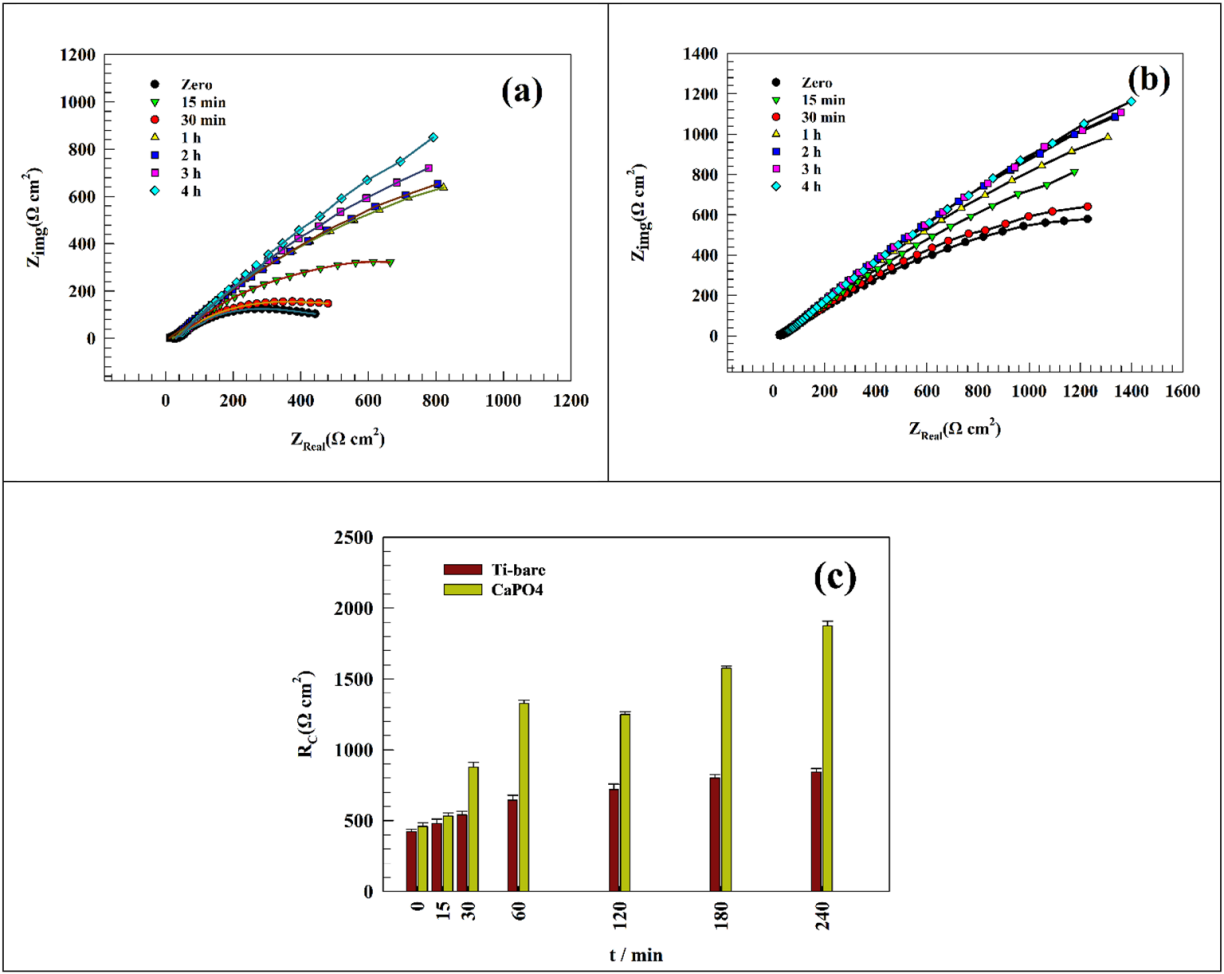
Representing of the Nyquist plot of the (**a**) Ti-bare and (**b**) Ti/CaPO_4_ after soaking in saliva for 240 min. (**c**) Comparison between the charge transfer resistance at different time intervals.

Figure 6a,b shows the Nyquist plot of the Ti-bare and Ti/CaPO_4_ surface through 240min. As represented in Fig. 6a, the Nyquist plot of Ti-bare in saliva solution shows the increase of resistance by time of soaking. As appeared in Fig. 6b, the value of the impedance increases in the resistive component, which indicates that the CaPO_4_ coat enhances the corrosion resistance by improving the surface compatibility^74^.

A comparison between the charge transfer resistance, calculated over different time intervals for Ti-bare and Ti/CaPO_4_ electrodes, was represented in (Fig. 6c). Where the R_ct_ values for Ti-bare are less than Ti/CaPO_4_ values, this explains the role of CaPO_4_ in enhancing the corrosion resistance between the coating and Ti surface in saliva solution. Additionally, the fitting parameters of Ti-bare and modified Ti/CaPO_4_ by using the fitting circuit appeared in Fig. 4, are represented in Tables 2 and 3. From this Table, we can deduce the many important features as the resistance of Ti-bare redouble from 424 Ω to 844 Ω after 240 min (Table 2), while for Ti/CaPO_4_ it 4 times multiplied during the same time, where the resistance increases from 460.08 to 1875.3 Ω (Table 3). Also, the high-value R_s_ values of Ti-bare than Ti/CaPO_4_ samples refers to the role of CaPO_4_ to decrease the R_s_ values during the time of immersion, which means that the good migration of the ions to the surface. The small capacitance value C points to the low capability of the surface to store charge. This result supports the good corrosion resistance of the surfaces where the capacitance is inversely proportional to the resistance^70^.

**Table 2:** Representation of the fitting parameters of Ti-bare electrode for 240 min in simulated saliva solution.

Immersion time (min)	Rs	Rct	C	w
	Ω cm^2^	Ω cm^2^	(F)	Y_o_
Zero	25.3	424	0.0019465	0.0011911
15	22.98	479	0.0022877	0.0013932
30	21.85	541	0.0017624	0.001487
60	21.23	645	0.001549	0.0015886
120	13.65	721	0.0022474	0.0014037
180	31.21	801	0.0016422	0.0017883
240	29.51	844	0.0015989	0.0017496

**Table 3:** Representation of Ti/CaPO_4_ electrode fitting parameters for 240 min in simulated saliva solution.

Immersion time (min)	Rs	Rct	C	w
	Ω cm^2^	Ω cm^2^	(F)	Y_o_
Zero	20.31	460.08	0.0087754	0.00066173
15	18.75	534.9	0.0087735	0.00098218
30	18.34	878.4	0.0087121	0.00070918
60	23.96	1329.3	0.0081457	0.00106471
120	18.56	1250.1	0.0066522	0.00108867
180	18.21	1577.4	0.0083068	0.00104972
240	17.6	1675.3	0.0064027	0.00099656

**Figure 7:**
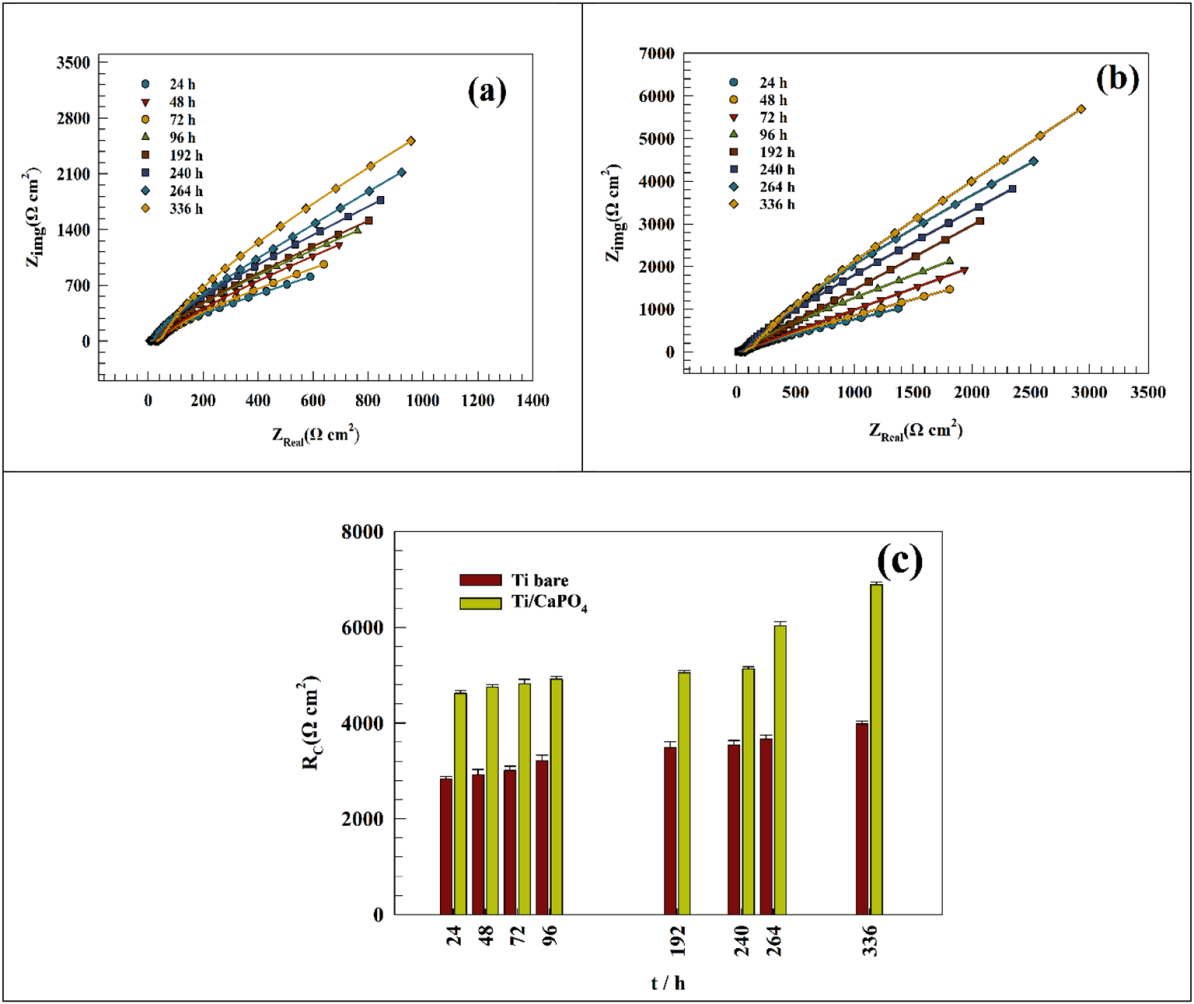
Representing of the Nyquist plot of the (**a**) Ti-bare and (**b**) Ti/CaPO_4_ after soaking in saliva for 336 h. (**c**) Comparison between the charge transfer resistance at different time intervals.

The effect of increasing the time of immersion from 240 min to 336 h or 14 days was studied in this section. As seen in Fig. 7a,b, the Nyquist plot of the Ti-bare and Ti/CaPO_4_ after 14 days of soaking in the saliva solution (without EO). The linear Nyquist plot indicates a non-charge transfer process, whereas the process is mainly diffusion^70^. Another time, the increase of the corrosion resistance of the Ti/CaPO_4_ sample reflects the positive impact of the CaPO_4_ layer, where the coating layer promotes surface durability. In Fig. 7c, a comparison between the charge transfer resistance over different time intervals for Ti-bare and Ti/CaPO_4_ electrodes was studied, and the results indicated that the charge transfer resistance reached a steady state after 14 days of soaking in the saliva solution. The resulting EIS fitting parameters based on the same fitting circuit (Fig. 4) were reported in the Tables 4 and 5. Where the resistance increases from 2831 to 3985 Ω for Ti-bare and from 4624 to 6890 Ω for Ti/CaPO_4_ during 336 h.

**Table 4:** Representation of the fitting parameters of Ti—bare electrode for 336 h in simulated saliva solution.

Immersion time (hrs)	Rs	Rct	C	w
	Ω cm^2^	Ω cm^2^	(F)	Y_o_
24	13.1	2831	0.0010499	0.0037738
48	27.2	2921	0.0010345	0.0036871
72	12.2	3013	0.0010318	0.0034524
96	7.3	3214	0.0010199	0.0032382
192	17.6	3487	0.0007478	0.0031025
240	11.9	3548	0.0005098	0.0020324
264	26.4	3672	0.0004855	0.0020198
336	28.8	3985	0.0003057	0.0020054

**Table 5:** Representation of Ti /CaPO_4_ electrode fitting parameters for 336 h in simulated saliva solution.

Immersion time (hrs)	Rs	Rct	C	w
	Ω cm^2^	Ω cm^2^	(F)	Y_o_
24	13.7	4624	0.0013603	0.0023142
48	6.9331	4752	0.0017779	0.0010329
72	13.713	4823	0.0009519	0.0014964
96	18.326	4919	0.0010911	0.0013919
192	14.229	5052	0.0010391	0.0014489
240	13.241	5137.5	0.0011269	0.0018313
264	18.21	6033.2	0.0007816	0.0014469
336	11.45	6890.2	0.0008409	0.0013728

**Figure 8:**
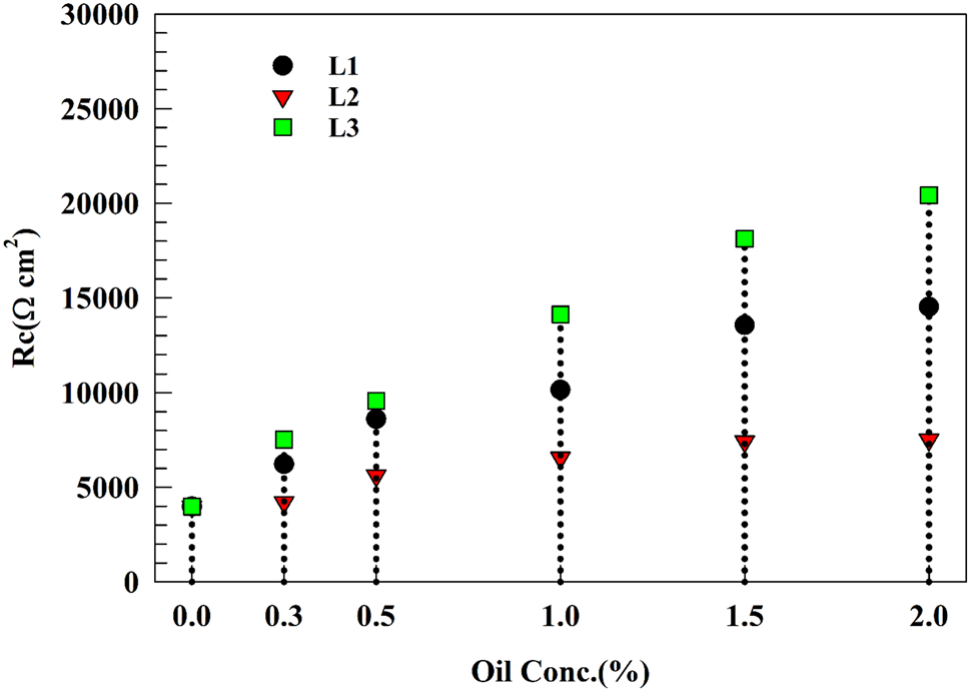
Comparison between the effect of essential oils concentration on Ti/CaPO_4_ and charge transfer resistance after soaking 336 h in saliva solution.

In this section, and for further enhancement of the corrosion resistance of the coated Ti/CaPO_4_ surfaces. Three EO were used for medical applications, named Cumin, Thyme, and Coriander oils (L1, L2, and L3). They injected into the operating solution. The effect of EO was studied over two periods, 240 min and 336 h, to compare the result of the same electrodes in the presence of the green extract. As represented in Fig. 8, the comparison between the effect of concentration of three EO on the charge transfer resistance Rct of Ti/CaPO_4_ appeared. Whereas different oil concentrations were used, i.e., 0.25% up to 2% (Wt/Wt). Rct of Ti/CaPO_4_ was observed to increase by 264.4, 88.2, and 437.5% for L1, L2, and L3, respectively, at 2% of EO concentration.

**Figure 9:**
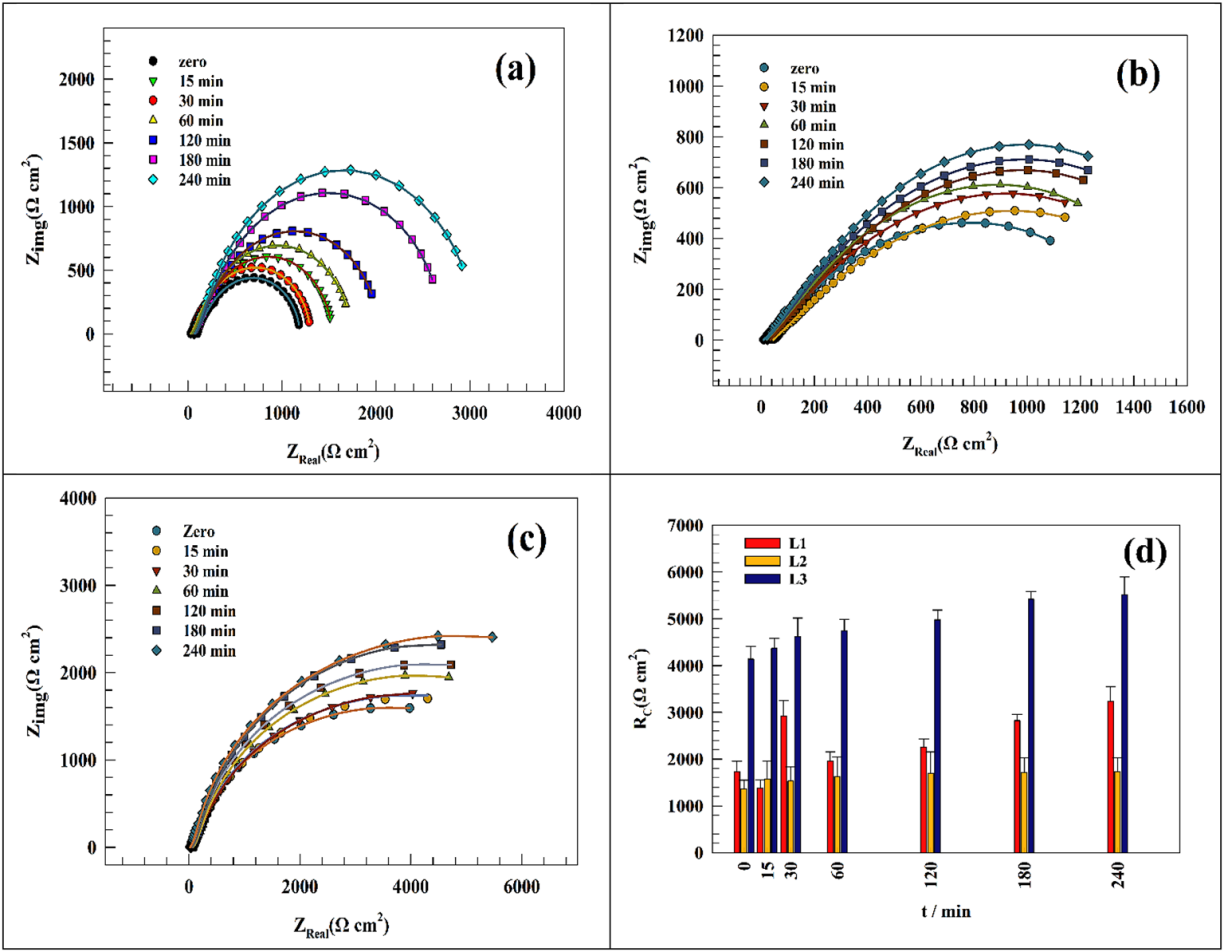
Representing of the Nyquist plot of the (**a**) Ti/CaPO_4_-L1, (**b**) Ti/CaPO_4_-L2, and (**c**) Ti/CaPO_4_-L3 after soaking in saliva for 240 min. (**d**) Comparison between the charge transfer resistance at different time intervals.

As illustrated in Fig. 9a–c, the Nyquist plot of modified Ti/CaPO_4_ electrode in saliva solution in the presence of 2.0% of different essential oils (L1, L2, and L3) for different time intervals up to 4 h. of soaking. However, the oil started diffusion with time, and the oil adsorption on the electrode surface enhanced the corrosion resistance over time. Hence, the diffusion of the saliva solution to the inner layers of the Ti sheet promotes the layer of the anodized Ti form, which is important for the corrosion resistance process. The progress of charge transfer resistance for each oil was followed as represented in Fig. 9d. By fitting the EIS result based on the fitting circuit (Fig. 4) for the Nyquist data, the Rct of the electrodes increased by 85, 27, and 33% for L1, L2, and L3, respectively. The change in the charge transfer value reflects the different abilities of an oil to be adsorbed on the Ti surface. Whereas the oil ingredients are different in chemical structures. The fitting parameters extracted from Nyquist plots are represented in Tables 6, 7 and 8.

**Table 6:** Representation of the fitting parameters of Ti/CaPO_4_ electrode for 240 min in simulated saliva solution in the presence of oil L1.

Immersion time (min)	Rs	Rct	C	w
	Ω cm^2^	Ω cm^2^	(F)	Y_o_
Zero	20.31	1278	0.00010635	0.00094051
15	32.95	1378	0.00012117	0.00080105
30	80.82	2928	0.00010347	0.00080572
60	28.32	1955	0.00099075	0.00088006
120	51.84	2256	0.00010146	0.00082697
180	79.44	2826	0.0001137	0.00078212
240	65.39	3234	0.00010993	0.00082703

**Table 7:** Representation of the fitting parameters of Ti/CaPO_4_ electrode for 240 min in simulated saliva solution in the presence of oil L2.

Immersion time (min)	Rs	Rct	C	w
	Ω cm^2^	Ω cm^2^	(F)	Y_o_
Zero	8.1	1363.5	0.00072305	0.0009921
15	21.3	1571.8	0.00088464	0.00096715
30	26.5	1531	0.00097682	0.00090402
60	34.74	1634	0.00095467	0.00093186
120	21.1	1695	0.00070791	0.00100214
180	31.4	1713	0.00083436	0.00094872
240	27.1	1734	0.00079157	0.0009859

**Table 8:** Representation of the fitting parameters of Ti/CaPO_4_ electrode for 240 min in simulated saliva solution in the presence of oil L3.

Immersion time (min)	Rs	Rct	C	w
	Ω cm^2^	Ω cm^2^	(F)	Y_o_
Zero	17.45	4140	0.00048172	0.00074208
15	38.16	4370	0.00057622	0.00066165
30	34.51	4621	0.00057219	0.00073426
60	34.12	4751	0.00060768	0.00067721
120	42.14	4982	0.00063483	0.00073281
180	31.41	5421	0.00060124	0.00073165
240	34.72	5520	0.00059045	0.00073124

**Figure 10:**
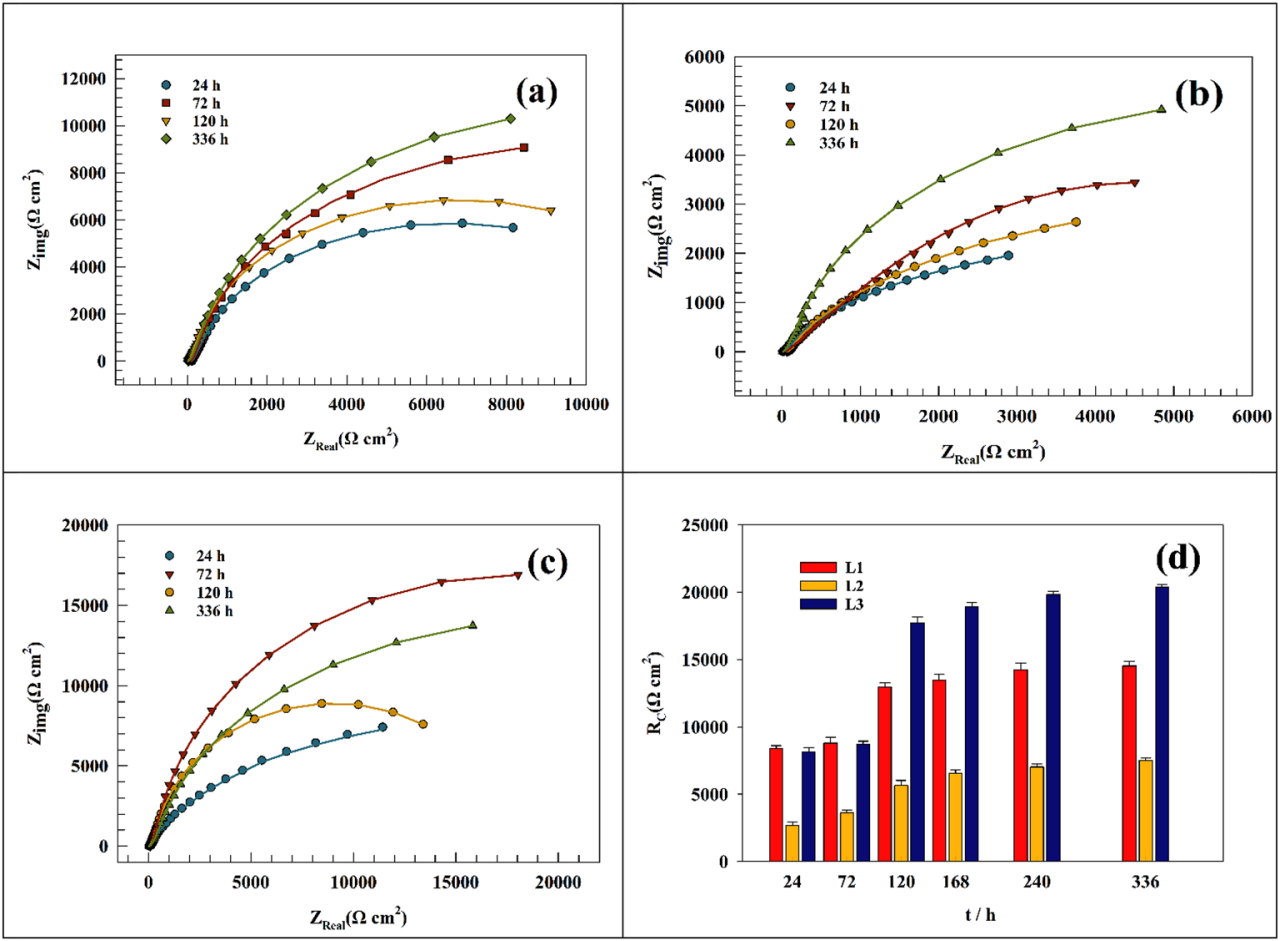
Representing of the Nyquist plot of the (**a**) Ti/CaPO_4_-L1, (**b**) Ti/CaPO_4_-L2, (**c**) Ti/CaPO_4_-L3 after soaking in saliva for 336 h (**d**) Comparison between the charge transfer resistance at different time intervals.

The modified electrode Ti/CaPO_4_ was investigated in saliva solution and injected oils for 14 days (see Fig. 10a,c). After the 336 h of soaking of Ti/CaPO_4_ in saliva, the electrode resistance started to reach the steady state where the charge transfer resistance was slightly changed. In the last 7 days, the resistance changed by 1.9, 7, and 2.8% for L1, L2, and L3, respectively. The anticorrosion activity of the EO was in the order of L3 > L1 > L2. The steric hindrance of the active component in the essential oils plays an important role in corrosion inhibition. For L3 oil, the most common ingredient, linalool, is long-chain alcohol, which has a smaller molecular volume than the bulky Thymol group (L2). The bulky group around the oxygen atom in thymol decreases the ability of thymol to be adsorbed on the titanium surface and diffusion through the CaPO_4_ layer. As shown in Tables 9, 10 and 11, the EIS-relevant data were reported for different essential oils corresponding to the different time intervals. The progress of charge transfer resistance for each oil was shown in Fig. 10d**,** where the promising effect of L3 & L1 appeared and reached the high value after 72 h to be stable at 336 h Although the value of the corrosion resistance is nearly the same for both L3 & L1 at the beginning of soaking (8166.5 & 8405 Ω, respectively), it doubles more than twice in the case of the L3 (20421Ω), compared to one and half times in the case of L1 (14,523 Ω) after 336 h at the ending soaking. While the L2 oil increases the corrosion resistance with low value in comparison to L1& L3, the progress of the resistance tripled through the time of soaking from 2680 to 7525 Ω.

**Table 9:** Representation of the fitting parameters of Ti /CaPO_4_ electrode for 336 h in simulated saliva solution in the presence of oil L1.

Immersion time (h)	Rs	Rct	C	w
	Ω cm^2^	Ω cm^2^	F	Y_o_
24	20.31	8405	0.00014939	0.0012252
72	10.12	8810	0.00012538	0.001392
120	18.34	12,954	0.00015158	0.0013833
336	23.96	14,523	0.00013158	0.0010647

**Table 10:** Representation of the fitting parameters of Ti/CaPO_4_ electrode for 336 h. in simulated saliva solution in the presence of oil L2.

Immersion time (h)	Rs	Rct	C	w
	Ω cm^2^	Ω cm^2^	F	Y_o_
24	10.45	2680.83	0.00026199	0.00098412
72	51.54	3618.72	0.00022871	0.0008737
120	40.52	5647.54	0.00024685	0.00033963
336	35.55	7525.19	0.00013158	0.0010647

**Table 11:** Representation of the fitting parameters of Ti/CaPO_4_ electrode for 336 h in simulated saliva solution in the presence of oil L3.

Immersion time (h)	Rs	Rct	C	w
	Ω cm^2^	Ω cm^2^	F	Y_o_
24	18.56	8166.5	0.00009636	0.0010891
72	19.92	8696.5	0.00010278	0.0010288
120	37.75	17,718	0.00009298	0.0010661
336	35.55	20,421	0.00009821	0.0010414

### Polarization technique

**Figure 11:**
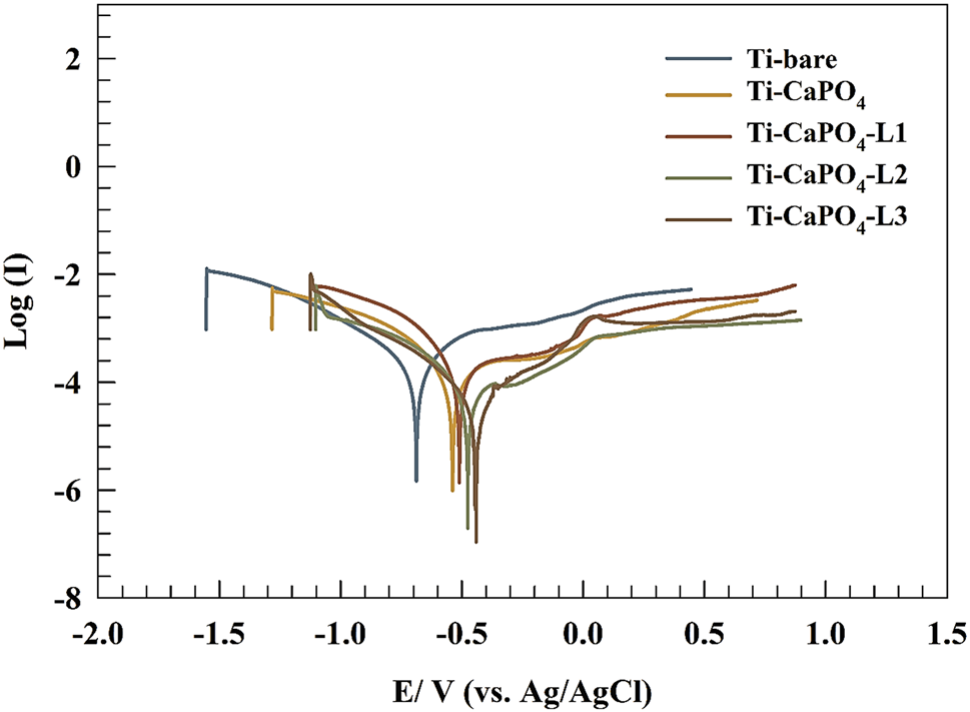
Polarization curves of different modified surfaces in saliva solution.

The Tafel technique was used to prove the best behavior of surfaces in saliva solution after 14 days of immersion. As represented in Fig. 11, the linear polarization curve of different modified electrodes Ti-bare, Ti/CaPO_4_, Ti/CaPO_4_-L1, Ti/CaPO_4_-L2, and Ti/CaPO_4_-L3 at a scan of 1 mV s^−1^ within potential window − 1 to 1 V vs. the open circuit potential. The linear polarization data can estimate the Tafel slopes (βa and βc). Furthermore, the values of corrosion current density (I_corr_) and corrosion potential (E_corr_) can be calculated by the interception of Tafel lines as listed in Table 12. The coated Ti/CaPO_4_ showed more positive corrosion current (E_corr_) values than the Ti-bare electrode, indicating that lower corrosion rate, which is less favored upon these surfaces. On the other hand, the corrosion current is a value that reflects the actual corrosion rate. The electrode with a higher corrosion current indicates a higher corrosion rate. The effect of essential oil addition was observed for the following modified surfaces: Ti/CaPO_4_-L1, Ti/CaPO_4_-L2, and Ti/CaPO_4_-L3. Whereas the E_corr_ of the modified electrode showed an order of L3 > L2 > L1, this result is equivalent to the result deduced from the EIS results. Furthermore, the polarization resistance reflects the current flow rate caused by electrochemical reactions. The sample with higher polarization resistance matched the sample with higher corrosion resistance. Also, the corrosion rates of each electrode were estimated using NOVA software based on the polarization data (See Table 12). The corrosion rate was expressed as mm of Ti foil per year (time interval). The electrode loss by corrosion in saliva fluid was Ti/bare > Ti/CaPO_4_ > Ti/CaPO_4_-L1 > Ti/CaPO_4_-L2 > Ti-CaPO_4_-L3 due to the adsorption of EO on modified Ti/CaPO_4_ surface.

**Table 12:** Represents the polarization parameters for Ti-bare, Ti/CaPO_4_, Ti/CaPO_4_-L1, Ti/CaPO_4_-L2, Ti/CaPO_4_-L3.

Electrode	${E }_{Corr}$(V)	${I}_{Corr}$(A/cm^2^)$\times {10}^{-5}$	Corrosion rate(mm/Year)	Polarization resistance
Ti-bare	− 0.68737	8.74	1.015919	298.0441
Ti/CaPO_4_	− 0.63884	7.25	0.640581	497.4933
Ti/CaPO_4_-L1	− 0.56587	5.28	0.613117	453.8515
Ti/CaPO_4_-L2	− 0.53073	4.62	0.520019	502.1876
Ti/CaPO_4_-L3	− 0.47607	3.47	0.403613	750.1955

## Conclusion

The findings of a recent study demonstrated that the development of a titanium surface for use in dental implant applications is not solely dependent on the type of surface modification used, but also on other aspects that may synergize with the modified materials to increase the implant's stability in the oral environment. The following is a reasonable inference to make considering the current results:

1. Anodization is still a simple electrochemical method used to treat the Ti surface to be active for further coating via the formation of a TiO_2_ thick layer.
2. Anodized Ti-bare sample has high corrosion resistance, especially after 336 h of immersion in saliva solution where it increases from 844 to 3985 ohms.
3. ACP-NPs were used to prepare nano flaks structure from the CaPO_4_ coat on the Ti implant.
4. The prepared Ti/CaPO_4_ sample is considered a good bioactive surface. It increases the corrosion resistance from 460 to 1675 Ω after 240 min and to 6890 Ω after 336 h of immersion in saliva solution.
5. Selected three EOs added to saliva solution, Cumin, Thyme, and Coriander, synergized with the CaPO_4_ coat on the Ti surface to enhance its stability by increasing the corrosion resistance to new high values equal to 14,523, 7525, and 20,421, respectively, after 336 h. of immersion in saliva solution.

Coriander, Thyme, and Cumin were found to have the most synergistic impact when stacked in that sequence. As a result, the chemical structure of the substance found in the oil might be utilised to explain the order in which the ability to preserve Ti/CaPO4 is ranked. Coriander has a chemical that has a linear structure, and this structure improves the capacity of ions to diffuse through the CaPO4 layer, which in turn raises the corrosion resistance. Despite the cumin compound's bulky structure's ability to restrict diffusion, the compound's resistance to corrosion actually reduced.

## Data Availability

The datasets used and/or analyzed during the current study are available from the corresponding Author on reasonable request.
